# Taxonomic investigation of the zooplanktivorous Lake Malawi cichlids *Copadichromis mloto* (Iles) and *C. virginalis* (Iles)

**DOI:** 10.1007/s10750-022-05025-1

**Published:** 2022-10-27

**Authors:** G. F. Turner, D. A. Crampton, B. Rusuwa, A. Hooft van Huysduynen, H. Svardal

**Affiliations:** 1 School of Natural Sciences, Bangor University, Bangor, Gwynedd LL57 2UW UK; 2 Vertebrates Division, Natural History Museum, Cromwell Road, London, SW7 5BD UK; 3 Department of Biology, Chancellor College, University of Malawi, Zomba, Malawi; 4 University of Antwerp, Universiteitsplein 1, 2610 Wilrijk, Belgium; 5 Naturalis Biodiversity Center, 2300 RA Leiden, The Netherlands

**Keywords:** Cichlid fish, Lake Malawi, Utaka, Systematics, Whole-genome sequencing, Geometric morphometrics

## Abstract

The taxonomic status of the zooplanktivorous cichlids *Copadichromis mloto* and *C. virginalis* has been confused since their original descriptions by lles in 1960. Whilst two forms of *C. virginalis*, ‘Kaduna’ and ‘Kajose’, were distinguished in the type material, *C. mloto* has not been positively identified since its original description. Here we re-examined the types as well as 54 recently collected specimens from multiple sampling locations. Genome sequencing of 51 recent specimens revealed two closely related but reciprocally monophyletic clades. Geometric morphological analysis indicated that one clade morphologically encompasses the type specimens of *C. virginalis* identified by Iles as the Kaduna form, including the holotype, whilst the other clade encompasses not only the paratypes identified as the Kajose form, but also the type series of *C. mloto*. Given that all three forms in Iles’s type series are from the same locality, that there are no meristic or character states to differentiate them and that there are no records of adult male *C. mloto* in breeding colours, we conclude that the Kajose form previously identified as *C. virginalis* represents relatively deeper bodied sexually active or maturing individuals of *C. mloto*.

**Supplementary Information:**

The online version contains supplementary material available at 10.1007/s10750-022-05025-1.

## Introduction

The Lake Malawi haplochromine cichlids represent the most species-rich vertebrate adaptive radiation known, comprising around 800 species (Konings, 2016) rapidly evolved from a common ancestor in a single lake (Malinsky et al., 2018; Svardal et al., 2020), and as such they represent a particularly difficult taxonomic challenge (Snoeks, 2004). The utaka are a group of zooplankton feeding cichlids that are both ecologically significant and commercially important as a food fish (Turner, 1996). Most utaka species are currently assigned to the genus *Copadichromis*, characterised by their relatively small, highly protrusible mouths and numerous long gill rakers (Eccles & Trewavas, 1989; Konings, 2016). They are generally silvery, countershaded and carry several dark spots on their flanks, although this is obscured in the reproductively active males that are conspicuously dark blue or black (Konings, 2016). A few species, known as ‘pure utaka’ lack flank spots entirely (Iles, 1960). The status of these has been confused for over 60 years, since the descriptions of *Copadichromis mloto* (Iles, 1960) and *Copadichromis virginalis* (Iles, 1960), both described from material collected at Nkhata Bay in the middle of the western shore of the lake. The former species was distinguished from the latter by its more slender build, but all other morphometric traits and all meristic counts overlapped (Iles, 1960). Whilst the original descriptions presented information on male breeding characteristics (often a key feature for discriminating closely related cichlid species) for *C. virginalis*, none were given for *C. mloto* which all appeared to be spent or reproductively inactive individuals. In the intervening years, no breeding adults of *C. mloto* were positively identified in collections, although Konings (2016) illustrated specimens proposed to be *C. mloto* that resemble those commonly assigned to *C. virginalis* in commercial trawl catches (Turner, 1996), but which had been assigned as *C. mloto* in earlier publications (e.g. Axelrod & Burgess, 1986). Further confusing matters, Iles’s original description of *C. virginalis* discussed two distinct sympatric forms which he recorded local fishermen referring to as ‘Kaduna’ (including the holotype) and ‘Kajose’ (included in the type series). Subsequent authors (e.g. Turner, 1996; Konings, 2016) suggested that these may be different species—a possibility tentatively discussed by Iles. In the course of a wider investigation of the Lake Malawi cichlid fauna, we were able to obtain a large number of specimens of ‘pure utaka’ from a number of locations in 2016–2017, which we used to investigate the status of *C. mloto* and *C. virginalis* using a phylogeny constructed from whole-genome sequences, coupled with geometric morphometric comparisons with Iles’ type material.

## Methods and materials

This study was based on the type material of *Haplochromis mloto* and *H. virginalis* examined and photographed in the Natural History Museum in London, along with 54 specimens collected from various locations around Lake Malawi in 2016–2017. The types of *H. virginalis* were classified by Iles into Kaduna and Kajose forms: these were separately catalogued: the holotype is a female Kaduna morph. During our examinations, external morphology suggested that two of the specimens were misclassified (perhaps through a mix-up by a later researcher), with the 88.3 mm SL individual in jar labelled as Kaduna looking more like a Kajose and the 96.5 mm SL Kajose looking more like a Kaduna. We used the re-classified identities in our analyses.

The freshly collected specimens were purchased from fishermen, and if not already dead, euthanised with anaesthetic overdose (clove oil); the right pectoral fin was cut off and placed in a vial of pure ethanol; the specimen pinned, labelled and photographed before being preserved in formalin after rigor mortis had set in, before being washed and preserved in ethanol. Morphometric analysis was based on digital analysis of the field photographs, along with photographs taken of the type material. Previous studies had examined meristics and morphological character states and did not find any diagnostic differences between the species (Iles, 1960; Eccles & Trewavas, 1989). Iles (1960) suggested that *C. mloto* had smaller teeth than similar species, but this was not supported by preliminary investigations—across species, large adult males were found to have strong simple teeth and whilst smaller fish had relatively smaller teeth, generally bicuspid in the outer row and tricuspid in the inner rows. This was not investigated further.

**Fig. 1:**
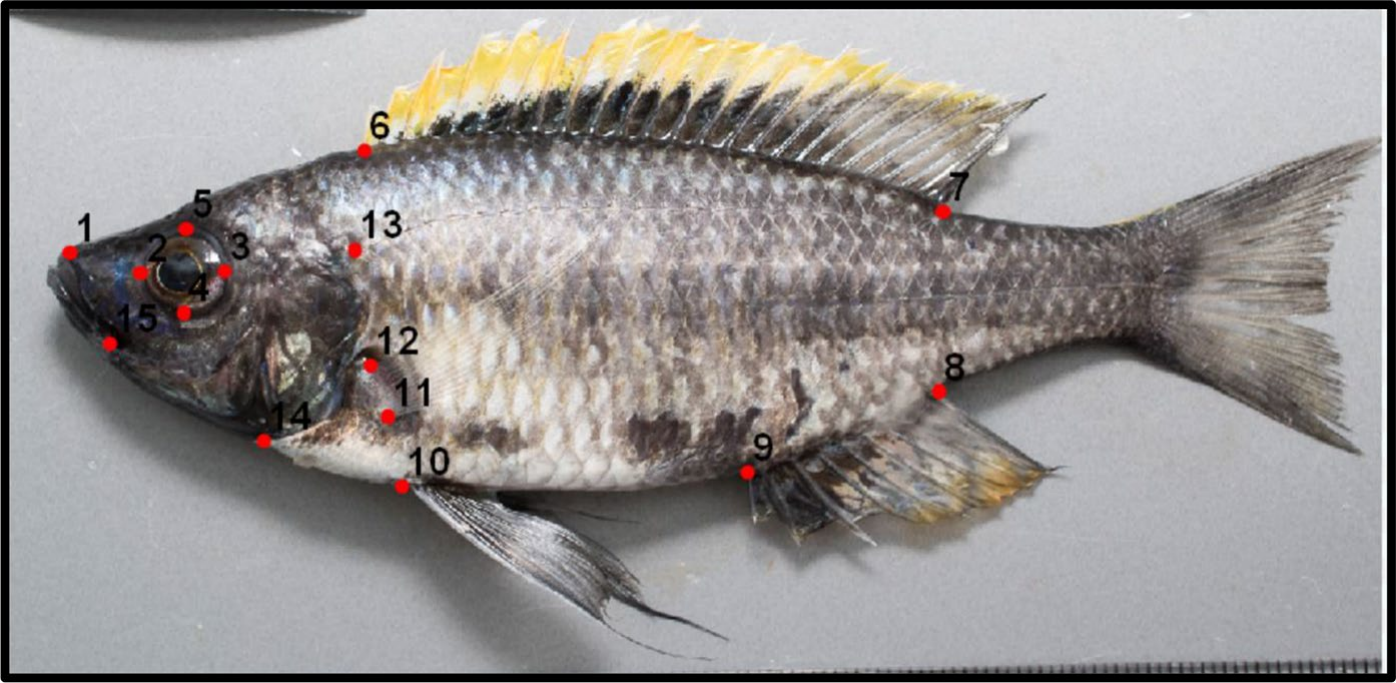
Landmarks used in Geometric Morphometric analysis of external body proportions (Specimen D12I04, *C. mloto*). (1) anterior tip of upper surface of maxilla (2) posterior reach of the eye (3) anterior reach of the eye (4) ventral reach of the eye (5) dorsal reach of the eye (6) anterior insertion point of dorsal fin (7) posterior insertion point of dorsal fin (8) posterior insertion point of anal fin (9) anterior insertion of anal fin (10) anterior/dorsal insertion of pelvic fin (11) anterior/ ventral insertion of pectoral fin (12) dorsal insertion of pectoral fin (13) point lateral line meets operculum (14) ventral-posterior extreme of preoperculum (15) anterior reach of the premaxillary groove

For geometric morphometric (GM) analysis, tpsUTIL (Rohlf, 2004) was used to build a file from scaled photographs, co-ordinates were recorded by tpsDig2 ver 2 (Rohlf, 2015) using the landmark tool, and provided with scale factors. We selected 15 homologous landmarks on the full specimen (Fig. 1). A CVS file containing the x and y coordinates of the landmarks for each specimen was then created and imported into MorphoJ (Klingenberg, 2011). Before Principal Components Analysis (PCA) was run on the geometric morphometric data, a generalised Procrustes analysis (GPA) was applied on the landmark data, to mathematically remove non-shape variation (Bookstein, 1989; Rohlf & Slice, 1990; Parés-Casanova et al., 2020). This was intended to eliminate morphological variation resulting from the size, position or orientation of the specimens. Then, a covariance matrix was generated from the resulting Procrustes shape coordinates and lastly the PCA was carried out. The resulting PC scores and centroid sizes were then imported into IBM SPSS Statistics 27, and One-Way Analysis of Variance used to test for group differences amongst component scores and centroid sizes (CS: a measure of overall body size) amongst the following 5 groups: types of *H. mloto*, types of *H. virginalis* (Kaduna), types of *H. virginalis* Kajose, sequenced *C. mloto* and sequenced *C. virginalis*. Post hoc tests (Tukey) were used to identify significant differences amongst groups, after simultaneous Bonferroni correction. Correlations between CS and PC scores were also calculated to aid interpretation.

DNA libraries for the above specimens collected in 2016–2017 as well as a single individual of the outgroup *Copadichromis chrysonotus* (Boulenger, 1908) were created and DNA was extracted from preserved fin clips using Qiasymphony DNA tissue extraction kits or PureLink ® Genomic DNA extraction kits and samples were whole-genome sequenced on an Illumina HiSeqX platform (150 basepair paired-end) to individual coverages between 13.8-fold and 42.1-fold (median of 17.1 -fold). Reads were aligned to the *Astatotilapia calliptera* reference genome fAstCal1.2 (GCA_900246225.3 https://www.ncbi.nlm.nih.gov/assembly/GCF_900246225.1) using BWA-MEM (BWA version 0.7.17) with default settings. Before reads were passed to BWA-MEM, we removed reads that did not pass platform/vendor quality controls by running command `samtools view -F 0 × 200 readfile.cram`. Alignments were further processed with `samtools fixmate -m` to fix mate information and sorted with `samtools sort`; duplicates were marked with `samtools markdup`. The variant site detection was performed with bcftools (version 1.14, Danecek et al., 2021). Any sites where more than 10% of mapped reads had a mapping quality zero, or where the overall mapping quality was less than 50 were masked. Sites for which mapping quality was significantly different between the forward and reverse strand (P < 0.001) were also masked, as well as sites where the sum of overall depth for all samples was unusually high (> 97.5 percentile) or low (< 2.5 percentile). Heterozygous sites for which a binomial test showed a significantly biased read depth of the reference and alternative alleles (PHRED score > 20) were also masked, representing between 0.02% and 0.15% of heterozygous sites per individual. Additionally, filters were applied to sites with excess heterozygosity (InbreedingCoeff < 0.2) and sites with > 20% missing genotypes. Only single nucleotide polymorphisms (SNPs) were retained for genetic investigation. After filtering, 17,307,336 biallelic SNPs across 22 chromosomes remained. Three specimens, D20E07, D20F05 and D20F06 collected at Lake Malombe were excluded from genetic analysis because of possible cross-contamination in the sequencing data apparent from substantial excess heterozygosity.

Pairwise sequence differences were calculated in 100 kb windows and averaged over the two haplotypes of each individual with a custom script based on scikit allel (available at https://github.com/feilchenfeldt/pypopgen3). To reconstruct genome-wide phylogenetic relationships, we summed pairwise genetic differences across all windows and constructed a neighbour-joining (NJ) tree using the Biophyton 1.79 Phylo package. We note that NJ has been shown to be a statistically consistent and accurate species tree estimator under incomplete lineage sorting (Rusinko & McPartlon, 2017), which is known to be pervasive amongst Malawi cichlid lineages (Malinsky et al., 2018). The resulting NJ tree was rooted using the *C. chrysonotus* sample as an outgroup. We implemented a block bootstrap by resampling with replacement the distance matrices of 100 kb windows 1000 times, each sample of a size corresponding to the total number of matrices (8,519), computed an NJ tree for each of these samples, and tested in what percentage of the samples the topology of a given node of the original tree was present (not shown: structure is identical to following tree). Finally, we also constructed a “split-tree” of samples where for each pairwise comparison the average within-individual heterozygosity (i.e. the pairwise sequence difference between the two haplotypes for each individual) was subtracted from the between-individual pairwise differences before constructing an NJ tree. We used the resulting NJ tree to obtain and estimate the split time between *C. virginalis* and *C. mloto*, by averaging the distance of present-day samples to the node separating the two species, using the previously inferred Malawi cichlid mutation rate of 3.5 × 10^–9^ (confidence intervals: 1.6–4.7 × 10^–9^; Malinsky et al., 2018), the accessibility mask from our variant calling, and assuming a generation time of three years. We note that this estimate of split time assumes constant effective population sizes and mutation rates.

**Fig. 2:**
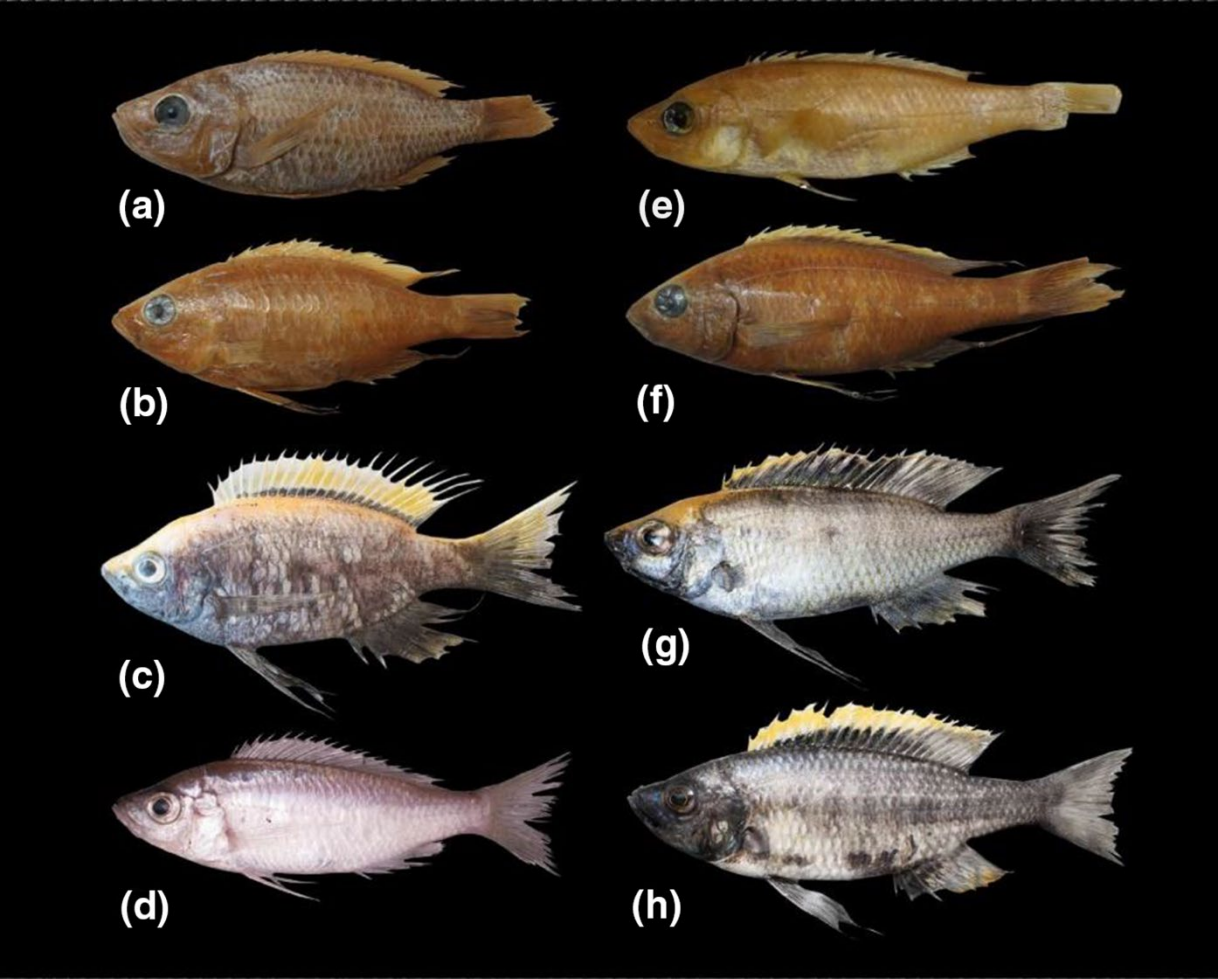
*Copdichromis virginalis*: **a** BMNH 1962.10.18.21, holotype of *H. virginalis*, female 79.8 mm SL; **b** BMNH 1962.10.18.22-30, paratype of *H. virginalis* (Kaduna morph), male; **c** D02G09, male, Nkhata Bay; *Copadichromis mloto*: **d** D17A03, female, Palm Beach, SE Arm; **e** BMNH 1961.10.18.31, holotype of *H. mloto*: apparent female 92.4 mm SL; BMNH 1961.10.18.40-50; **f** paratype of *H. virginalis* (Kajose morph), male; **g** D06E04, male, Chiweta Beach, Chilumba; **h** D12I04, male, trawled from 30 to 40 m depth, SW of Makanjila

Specimens examined:

A representative selection of specimens is shown on Fig. 2. D-codes are those of our sample collection and can be matched to specimens, photos and tissue samples.

*Copadichromis mloto* (Iles, 1960).

BMNH 1962.10.18.21 Holotype of *Haplochromis mloto*, coll. T.D.Iles, Nkhata Bay, Lake Nyasa 79.8 mm SL.

BMNH 1962.10.18.22-30 Paratypes of *Haplochromis mloto*, coll. T.D.Iles, Nkhata Bay, Lake Nyasa 9 specimens 76.8–114.7 mm SL.

BMNH 1961.10.18.40-50 Paratypes of *Haplochromis virginalis* (Kajose form), coll. T.D.Iles, Nkhata Bay, Lake Nyasa 10 specimens 70.4–111.2 mm SL.

BMNH 1961.10.18.32-39 Paratype of *Haplochromis virginalis* (Kaduna form), coll. T.D.Iles, Nkhata Bay, Lake Nyasa 1 specimen 107.5 mm SL.

D06E04 Chiweta Beach, Chilumba, 24-Feb-16, purchased from beach seiners, 1 specimen;

D12I04, I05, I08 Trawled from 30-40 m depth SW of Makanjila, 2 March 2016, 3 specimens;

D14E01-3 trawled from 40 m depth off Malembo, SW Arm, 4 March 2016, 3 specimens;

D17A01-D17A09, D17B03-D17B05, D17B07-B10 purchased from beach seiners, Palm Beach, SE Arm, 21 Jan 2017, 16 specimens;

D20E06-G02 purchased from Nkatcha fishermen, Lake Malombe, 24 Jan 2017, 17 specimens.

*Copadichromis virginalis* (Iles, 1960).

BMNH 1961.10.18.31 Holotype of *Haplochromis virginalis* (Kaduna form), coll. T.D.Iles, Nkhata Bay, Lake Nyasa 92.4 mm SL.

BMNH 1961.10.18.32-39 Paratypes of *Haplochromis virginalis* (Kaduna form), coll. T.D.Iles, Nkhata Bay, Lake Nyasa 5 specimens 83.9–98.1 mm SL.

BMNH 1961.10.18.40-50 Paratypes of *Haplochromis virginalis* (Kajose form), coll. T.D.Iles, Nkhata Bay, Lake Nyasa 1 specimen 96.5 mm SL.

D02G08-I01 purchased from fish traders, Nkhata Bay 21 Feb 2016, 14 specimens.

## Results

**Fig. 3:**
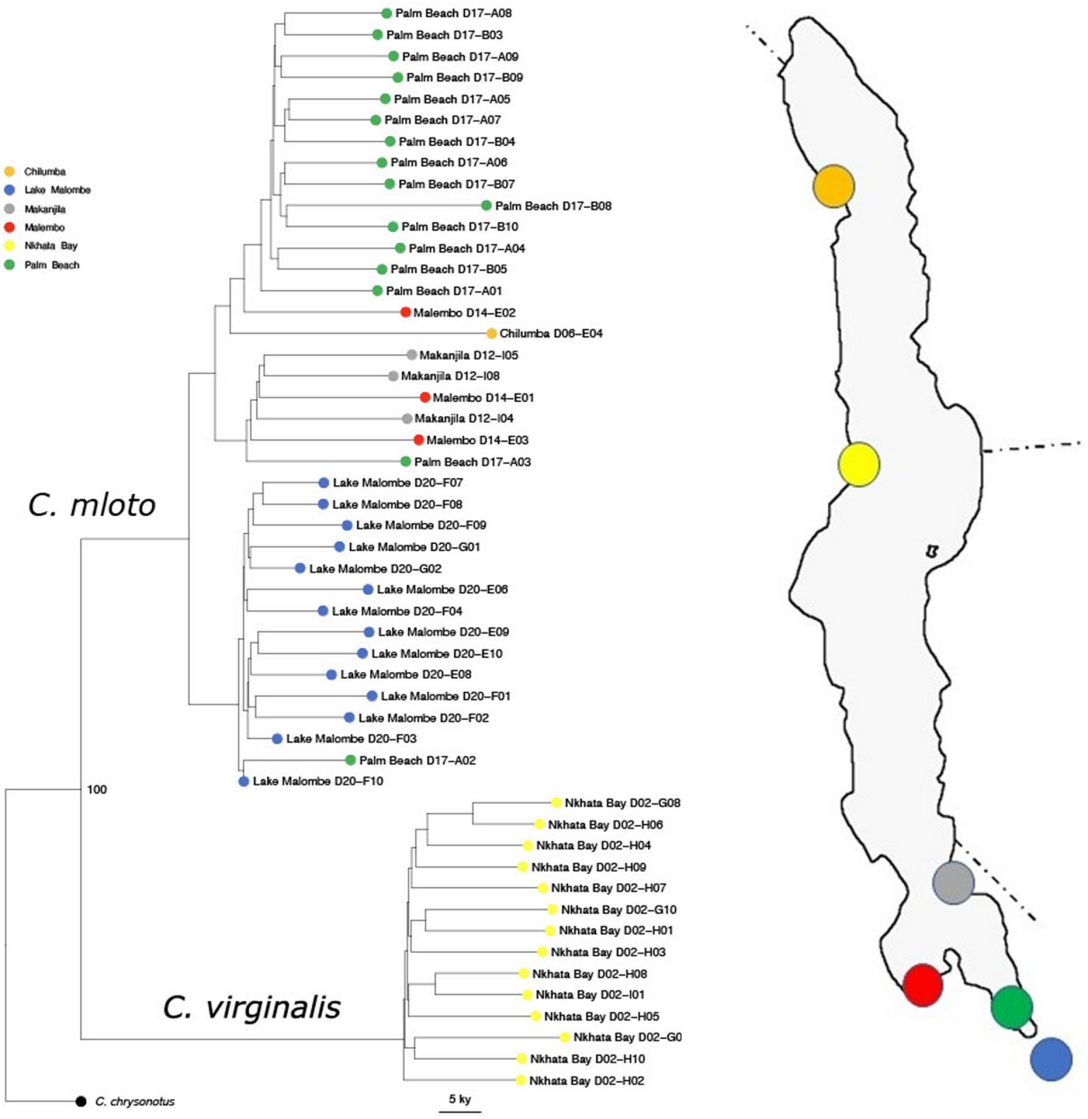
Neighbour joining ‘split tree’ based on 17 million SNPs from 51 whole-genome sequences of *Copadichromis virginalis /mloto* specimens collected in 2016–2017 from 6 localities in Lakes Malawi and Malombe. Code numbers refer to collection codes. Bootstrap support of 100% is shown for the divergence of *C. virginalis* and *C. mloto*. The tree was rooted on a specimen of *C. chrysonotus*

We first produced a neighbour-joining coalescent tree based on pairwise genetic differences at genome-wide SNP loci of the 51 recently sampled specimens (not shown). The tree showed relatively long terminal branches, consistent with the recent population separation and growth- because in this situation, between-population variation (internal nodes) will be small compared to individual within-population variation. However, it still clearly resolved a sister relationship between two groups representing the Nkhata Bay specimens and all other ‘pure utaka’ specimens. Block-bootstrap resampling in 100 kilobase windows confirmed 100% bootstrap support for the reciprocal monophyly of these groups. That said, only 3.5% of local gene trees supported this separation across all samples, an expected result given the observation that incomplete lineage sorting is widespread across Malawi cichlids (Malinsky et al., 2018). The split tree yielded a similar topology to the coalescent tree, again with 100% bootstrap support for the node separating the two clades (Fig. 3) with an estimated split time of the two clades at 53 kya (mutation rate based confidence interval 40–115 kya).

All of the Nkhata Bay specimens collected freshly in 2016 were adult males with a very similar colour phenotype (Fig. 2c): dark grey body, pelvic, anal and caudal fins, with a bright yellow upper surface extending from the tip of the snout to the caudal fin. The dorsal fin was yellow with a narrow black stripe on the base, extending sharply upwards to the tip of the soft-rayed portion. The black stripe was sometimes bordered by white below the yellow area. Some of the paratypes of *H. virginalis* showed similar dark markings on the dorsal fin, particularly the 90.6 mm SL, 86.9 mm SL specimens, labelled as ‘Kaduna’ (Fig. 2b), as is the female holotype (Fig. 2a).

The other clade comprised both males and females. Male breeding colour was also dark grey to black with yellow on the upper surface, but the distribution of the yellow pigment was much more restricted, sometimes to the upper part of the dorsal fin, sometimes to the upperpart of the head and nape, but never extending far behind the first few dorsal fin rays and even then, only as a very thin band. The black in the dorsal fin always formed a much wider band at the base, sometimes extending right to the tip of the fin along its entire length (Fig. 2g, h). A wider dorsal fin black band was also shown by several of the *C. virginalis* types labelled as Kajose form, particularly the 100.7 mm SL and 102 mm SL specimens (Fig. 2f). None of the *C. mloto* types showed any hint of male breeding colours (Fig. 2e), similar to freshly collected females (Fig. 2d): all were uniformly pale, slightly countershaded. Thus, male colours could not be used to characterise this species from the type material.

**Fig. 4:**
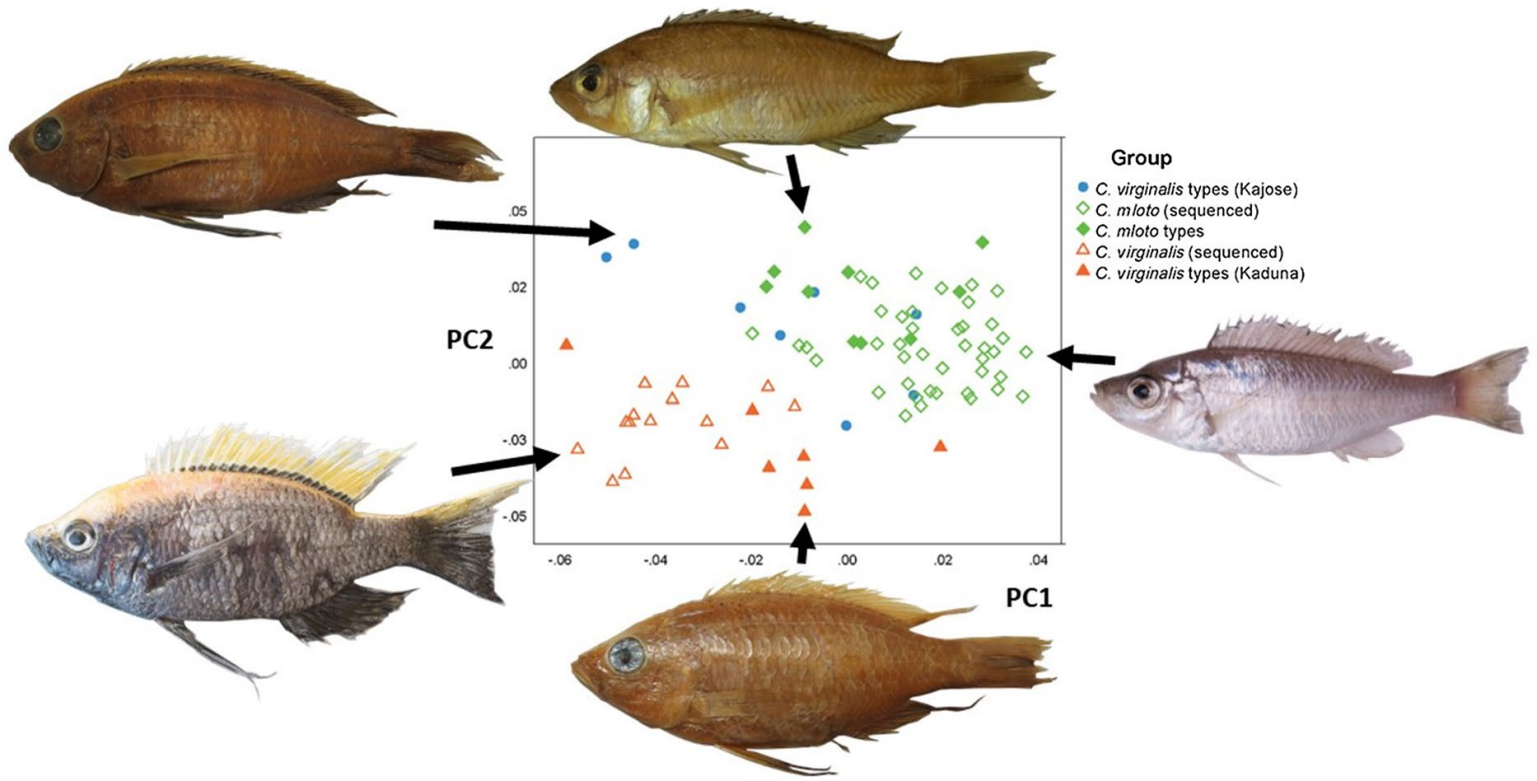
Above: Principal components analysis based on geometric morphometrics separates the *C. virginalis* specimens (red, types shown by filled triangles) from the *C. mloto* (blue, types filled circles, types of *H. virginalis* Kajose morph green filled diamonds). Separation is on a combination of the two major components, PC1 and PC2. Examples of extreme phenotypes on each component are shown, with the slender female/immature *C. mloto* occupying the upper right sector, and the deep-bodied mature male *C. virginalis* the lower left area, with deeper-bodied male *C. mloto* the upper left. Higher PC2 scores seem to be associated with a shorter snout and straighter upper head profile

The first five principal components were responsible for 71% of the sample variance (see Table 1). Overall, the two species were separated fairly clearly on a plot of the first 2 principal components, in an additive fashion (Fig. 4). Analysis of variance showed that all six comparisons between groups identified as *C. mloto* v *C. virginalis* were significantly different in body shape, including the Kaduna and Kajose (= *C. mloto*) types of *C. virginalis*, which both comprised a mix of sexes and which did not differ significantly in body (centroid) size. Significant differences were also found in two of the four within-species comparisons. The difference between the Kaduna types of *C. virginalis* v the sequenced specimens in PC2 may have been the result of sexual dimorphism, as all the sequenced specimens were male, whilst the types were mixed sex. The difference between the sequenced *C. mloto* and the Kajose types may have been influenced by residual allometric effects as these two groups differed significantly in centroid size. Overall, in our sample, *C. mloto* specimens had a smaller average size (mean centroid size ± 95% confidence interval: 8.5 ± 0.4) than those of *C. virginalis* (10.5 ± 0.2) suggesting that allometries may also have influenced overall geometric morphometric differences. A GLM of PC score v centroid size, assuming equality of slopes, indicated that PC1 was significantly correlated with centroid size (*P* < 0.001) but PC2 was not (*P* = 0.106). When we restricted the analysis to specimens of centroid size > 10, we obviously lost some statistical power (total df = 26 v 80 for the full sample), and in the reduced dataset, PC1 was marginally no longer significantly different amongst species (*P* = 0.061), but PC2 remained highly significant (*P* < 0.001). PC5 was also significantly different. Therefore, we believe that shared allometries and differences in average size explain some, but not all, of the species differences in GM analysis and that there is size-independent morphological differences between them.

## Discussion

The identification of *C. mloto* and *C. virginalis* has been confused since the original descriptions by Iles in 1960. Our initial field classification was prompted by observation of differences in male breeding dress and body shape of the fresh Nkhata Bay material (all collected together in 2016) compared to the other specimens in the 2016–17 collections, later confirmed to represent *C. virginalis* and *C. mloto*, respectively.

Fryer & Iles (1972, p. 545) reported differences in the breeding seasons between the species, *C. virginalis* breeding from May to June, whilst *C. mloto* bred from August to November. This makes it all the more puzzling that the original description contained no information on the breeding colour of *C. mloto* males, and no breeding males were included in the type series. They also reported *C. virginalis* and *C. mloto* (along with ‘*Haplochromis kajose’*!) were often found together in mixed species shoals (p. 295). Axelrod & Burgess (1986, but originally published in 1973 and periodically republished with similar/identical text but extra photos) showed a male and female *C. mloto* correctly identified and a female *C. virginalis* with very orange pelvic and anal fins, which may be *Mchenga flavimanus* (Iles, 1960) or *Copadichromis cyaneus* (Trewavas 1935). Fisheries data collection in the Monkey Bay research unit identified *C. mloto* as a common species in trawl catches in the southern arms of the lake (see Eccles & Trewavas, 1989), but this was later contradicted by Konings (1990), Turner (1996) and Snoeks & Hanssens (2004) who identified this species as *C. virginalis*.

Our combination of morphological investigation and sequencing of more recently collected material provides us with strong evidence that Iles’ Kajose form of *C. virginalis* is in fact conspecific with *C. mloto*, with the former representing deeper-bodied specimens including mature males and ripe females, whilst the types of the latter are slender individuals including spent fish. Notably, none of the *H. mloto* type material shows any sign of male breeding colours. Final certainty about the genetic identity of the type specimens relative to recent samples could only be obtained from their sequencing, which would be challenging given their preservation in formalin, but might become possible in the future (e.g. see Hahn et al., 2021). For the time being, the results presented here support the original assessment by Eccles & Trewavas (1989) that most pure utaka specimens commonly found in trawl catches correspond to *C. mloto* as do the species illustrated on Konings (1990, p. 256) (middle photo), Turner (1996, p. 53) and discussed on pp. 67–69 as *C. virginalis.*

It is worth noting that the two male specimens shown by Turner (1996) show quite divergent breeding colours, as indeed do some of the specimens we collected, so it is quite possible that *C. mloto* as presently defined may represent a complex of species: resolving this would probably involve sampling males in breeding colour from Nkhata Bay—the type locality, but this has yet to be done. The specimens illustrated by Konings (2016, 324) as *C. mloto* do seem to correspond to our identification of that species. He reports males building bowers at depths of around 23 m off Otter Point in the south of the lake, and describes the species as mainly being found over sand. Turner’s (1996) records and the sampling for the present study indicate that *C. mloto* is abundant in Lake Malombe and is found in Lake Malawi from the shallowest trawls (18 m) down to 114 m. We consider that the specimens examined by Anseeuw et al. (2008, 2011) as *C.* sp’virginalis kajose’ largely or entirely correspond to *C. mloto*: certainly those from Lake Malombe and SE Arm of Lake Malawi.

Konings (2016) also discusses *C. virginalis* and illustrates a number of males in breeding dress from a number of sites around the lake. These all correspond reasonably well to *C. virginalis* as we now define it, showing the characteristic narrow black dorsal fin base with the sharp uptick in the soft-rayed region. His *C. virginalis* are from Higga Reef (p.153), on the eastern shore of the lake across from Nkhata Bay on the West, and from Manda (p.154), also on the east coast but much further north. These males have a similar colour pattern to our Nkhata Bay specimens, but the yellow colour is replaced by pale blue: these would seem to represent geographic variants of *C. virginalis*, or allopatric sister species. His *C.* sp. ‘virginalis kajose’ from Kirondo (far north east) looks very similar to the Manda and Higga Reef specimens (same colour and dorsal fin markings) and does not correspond to *C. mloto*/kajose. This is also true of his *C.* sp. ‘firecrest mloto’ and *C. ilesi* both from Gome on the south eastern part of the lake near the Malawi/Mozambique border- their affinities would seem to lie with *C. virginalis* and not *C. mloto*. For this reason, we suggest the former should simply be referred to as *C.* sp. ‘firecrest’. In the original description of *C. ilesi*, it is apparent that Konings (1999) believed he was describing *C. virginalis* Kajose (which we now identify as *C. mloto*), although he did not include the Iles type specimens in his type series, but just specimens he had collected from Gome, illustraing males with a blue blaze. We agree with Snoeks and Hanssens (2004) that these are not *C. mloto/* Kajose. In fact, photographs of *C. ilesi* from Gome in Konings (2016) look almost identical in colour to our *C. virginalis* (Kaduna) from Nkhata Bay, showing a male with a yellow blaze, and so perhaps *C. ilesi* might be best considered as a junior synonym of *C. virginalis*. However, not having examined the types of *C. ilesi* or sequenced specimens from Gome, we do not formally propose this.

The occurrence of two very differently coloured ‘*C. virginalis*’ forms at Gome—*C. ilesi* and *C.* ‘firecrest’ -suggests that some of these geographic colour forms are likely to deserve recognition as distinct species. The status of the populations illustrated by Konings (2016) as *C.* sp. ‘virginalis Chitande’ (NW coast around Chilumba) and *C.* sp. ‘virginalis gold’ (Nkanda, northeastern shore) is less clear. The former has pale blue upper parts and a weakly developed set of ‘virginalis’ black dorsal fin markings, whilst the latter has yellow upper parts, but no black in the dorsal at all. Irrespective of their status in relation to *C. virginalis*, we consider that none of these are referrable to *C. mloto*. Konings reports that these different variants of the *C. virginalis* complex all breed on rocky areas, constructing a bower consisting of a simple crater dug on a soft substrate underneath an overhanging rock, so that a complete circle is not produced, as the rock interrupts the circle. More certainty about the taxonomic status of different morphs will require more sampling and sequencing.

The estimated age of separation of the two species at around 50kya—albeit with substantial margin for error—is consistent with geological estimates for the last major refilling of the lake basin from around 100kya (see Scholz et al., 2007; Ivory et al., 2016). The strikingly long terminal branches observed in the coalescent NJ reflect that cross-sample coalescence in many regions of the genome pre-dates the estimated species split, indicating substantial shared ancestral variation and incomplete lineage sorting. This accurately reflects the challenge in constructing robust phylogenetic estimates in Malawian cichlids, even with full genome sequence information (Scherz et al., 2022). A factor influencing this pattern may be a rapid population expansion around the time of speciation, perhaps co-incident with colonisation of deep-water habitats (Genner et al., 2010), whilst extensive introgression amongst species is probable and will further confound phylogeny estimation (Scherz et al., 2022).

## Conclusion

On the basis of our morphological and genomic studies, we conclude that *Copadichromis mloto* is an abundant species in a variety of fisheries in Lake Malawi, including the commercial trawls in the southern arms and the Nkatcha seines in Lake Malombe. It lives largely over sand/mud bottoms and can be recognised from its male breeding colours, body morphology and genome sequences. It includes the form previously recognised as Kajose from the type series of *Haplochromis virginalis*. The true *Copadichromis virginalis* is more rock-associated, building bowers up against overhanging rocks and characterised by breeding males with an extensive ‘blaze’ of bright colour (yellow at the type locality) on the whole of the upper surface and a distinctive black line on the lower part of the dorsal fin, showing a sharp uptick in the soft-rayed portion. Each of these two species may consist of a number of populations or geographic races with slightly different male colours, some of which may deserve recognition as further species.

**Table 1:**
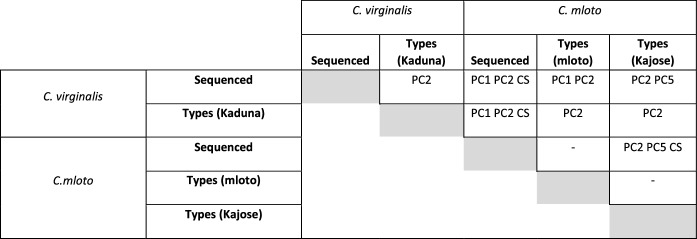
Morphological comparisons amongst 5 populations: analysis of variance with post hoc Tukey tests, with Bonferroni comparison for multiple testing, alpha < 0.005 was taken as significant

CS = centroid size

## Supplementary Information

Below is the link to the electronic supplementary material.

Supplementary file1 (DOCX 21 kb)
