# Supplementary material for: Taxonomic investigation of the zooplanktivorous Lake Malawi cichlids Copadichromis mloto (Iles) and C. virginalis (Iles)
Source: Hydrobiologia. 2022 Oct 27;850(10-11):2165–75. doi: 10.1007/s10750-022-05025-1 (PMC10261246; doi:10.1007/s10750-022-05025-1)
Supplement: Supplementary file 1 — Supplementary file1 (DOCX 21 kb) [file 10750_2022_5025_MOESM1_ESM.docx]

Appendix

Table 2: Summary of the Principal Components Analysis results, Geometric Morphometrics

| Variable | Eigenvalues  (x 10^-4^) | % Variance | Test among groups;  Anova F_4,76_= | Correlation with CS, Pearson’s r_81_= |
| --- | --- | --- | --- | --- |
| PC1 | 7.26 | 24.22 | 39.99; P<0.001 | -0.635; P<0.001 |
| PC2 | 5.73 | 19.11 | 28.99; P<0.001 | -0.265; P=0.017 |
| PC3 | 3.48 | 11.62 | 4.58; P=0.002 | -0.373; P=0.001 |
| PC4 | 2.95 | 9.83 | 3.68; P=0.009 | -0.122; P=0.028 |
| PC5 | 2.04 | 6.82 | 12.89; P<0.001 | -0.293; P=0.008 |
| Centroid Size | n/a | n/a | 26.59; P<0.001 | n/a |
